# Improved prediction and flagging of extreme random effects for non-Gaussian outcomes using weighted methods

**DOI:** 10.1093/biomtc/ujaf094

**Published:** 2025-07-26

**Authors:** John Neuhaus, Charles McCulloch, Ross Boylan

**Affiliations:** Department of Epidemiology and Biostatistics, University of California, San Francisco, CA 94143-0560, United States; Department of Epidemiology and Biostatistics, University of California, San Francisco, CA 94143-0560, United States; Department of Epidemiology and Biostatistics, University of California, San Francisco, CA 94143-0560, United States

**Keywords:** hierarchical model, predicted random effects, profiling

## Abstract

Investigators often focus on predicting extreme random effects from mixed effects models fitted to longitudinal or clustered data, and on identifying or “flagging” outliers such as poorly performing hospitals or rapidly deteriorating patients. Our recent work with Gaussian outcomes showed that weighted prediction methods can substantially reduce mean square error of prediction for extremes and substantially increase correct flagging rates compared to previous methods, while controlling the incorrect flagging rates. This paper extends the weighted prediction methods to non-Gaussian outcomes such as binary and count data. Closed-form expressions for predicted random effects and probabilities of correct and incorrect flagging are not available for the usual non-Gaussian outcomes, and the computational challenges are substantial. Therefore, our results include the development of theory to support algorithms that tune predictors that we call “self-calibrated” (which control the incorrect flagging rate using very simple flagging rules) and innovative numerical methods to calculate weighted predictors as well as to evaluate their performance. Comprehensive numerical evaluations show that the novel weighted predictors for non-Gaussian outcomes have substantially lower mean square error of prediction at the extremes and considerably higher correct flagging rates than previously proposed methods, while controlling the incorrect flagging rates. We illustrate our new methods using data on emergency room readmissions for children with asthma.

## 1 INTRODUCTION

Investigators often seek to accurately predict random effects from generalized linear mixed models (GLMM) fitted to clustered data in order to evaluate the performance of clusters such as physicians, hospitals, or geographic areas and identify or flag extreme performers. For example, Estes et al. (2020) sought to identify or flag dialysis facilities with high recurrent anemia events. Marafino et al. (2018) predicted significant changes in vital signs during an intensive care unit stay to later associate with in-hospital mortality. The California Surgeon Risk-Adjusted Operative Mortality Rates for Coronary Artery Bypass Graft Surgery (California Health and Human Services, 2023) reports overall risk-adjusted operative mortality rates for coronary artery bypass surgery for each of 276 surgeons in the state in 2017–2018. See McCulloch (2024) for these data and their analysis.

Much of the existing literature on predicted random effects focuses on prediction without regard to whether the random effect is extreme or not and on settings with continuous, normally distributed outcomes. However, prediction of unusual/extreme random effects is a common objective and the outcome of interest is often non-normal, particularly binary. In this paper, we consider a study of asthma-related visits to emergency departments (ED) in California with data clustered within zip codes (Bardach et al., 2022) where interest focuses on the prediction and identification of zip codes with high rates of asthma-related ED revisits. Pediatric asthma-related ED visit rates are a national quality measure for health plans, so provide a relevant exemplar accountability metric.

McCulloch and Neuhaus (2021) developed weighted prediction methods for linear mixed effects models with Gaussian outcomes that more heavily weight contributions from extreme random effects, identified several useful classes of weighting functions and showed that weighted predictors outperform standard methods in terms of mean square error of prediction (MSEP) for extreme random effects. McCulloch et al. (2024) extended the work of McCulloch and Neuhaus (2021) to develop methods to identify or flag extreme random effects in Gaussian outcome settings. In particular, McCulloch et al. (2024) developed flagging methods that they called self-calibrated that control the incorrect flagging rate using very simple flagging rules. The work of McCulloch and Neuhaus (2021) and McCulloch et al. (2024) took advantage of the fact that closed-form expressions for predicted random effects and probabilities of correct and incorrect flagging are available for Gaussian outcomes and dependence on model parameters takes a simple form. However, such closed-form expressions are not available for the usual non-Gaussian outcomes and dependence on parameters is more complicated than for the Gaussian case.

The work in this paper builds on previous work on prediction of random effects as well as the identification or “flagging” of extreme random effects for non-Gaussian outcomes (see review articles by Jones and Spiegelhalter (2011) and Normand et al. (2016)) but focuses on the prediction of extremes and the control of the incorrect flagging rate. Skrondal and Rabe-Hesketh (2009) presented Bayesian prediction methods appropriate for any generalized linear mixed model but these approaches do not focus on extreme random effects. Lyles and Xu (1999) studied flagging based on predicted random effects, but their investigations focused on just 3 predictors for Gaussian outcomes (the usual mixed model predictor, that is, the best linear unbiased predictor (BLUP), the fixed effects and constrained Bayes predictors) and they did not purposefully seek to control the incorrect flagging rate of these methods. Lyles et al. (2006) focused almost exclusively on the mean square error of prediction for alternative predictors but did propose a single method that controlled the incorrect flagging probability (their constrained specificity predictor, which they denote by “CSP”). They reported its use in an example, but did not systematically evaluate its performance. Kalbfleisch and Wolfe (2013) developed flagging methods appropriate for both Gaussian and survival outcomes and focused on random effects predictors they called “fixed effects” predictors which involve no shrinkage. However, fixed effects predictors are highly variable and can provide poor correct flagging rates compared to weighted predictors in linear mixed models (McCulloch, 2024). Furthermore, for binary outcomes, issues arise in even defining the fixed effects predictor when the observed proportions are 0 or 1 and fixed effects predictors can perform poorly when numbers of patients or events are small (Normand et al., 2016).

In this paper, we focus on the prediction of extreme random effects and develop numerical methods, as well as theory, to calculate weighted random effect predictors for non-Gaussian outcomes, including algorithms to calculate “self-calibrated” predictors and assess their performance with comprehensive numerical evaluation which includes comparisons to existing best prediction methods. We show that the new weighted methods provide more accurate prediction of extreme random effects and the weighted and self-calibrated predictors offer better control of incorrect flagging rates as well as improved identification of extreme random effects compared to standard best prediction methods. We illustrate our new methods using data on emergency room readmissions for children with asthma (a binary outcome) which also shows the advantages of the newly proposed methods.

## 2 STATISTICAL MODEL

We assume that responses $Y_{ij}$ follow a canonical link generalized linear mixed effects model (McCulloch et al., 2008), where $i=1, \ldots , m$ indexes clusters and $j=1, \ldots , n_i$ indexes units within clusters. Such models include the mixed effects logistic, Poisson, gamma, and familiar normal-theory linear mixed effects models as special cases and have conditional density for responses $y_{ij}$ of the form


(1) \begin{eqnarray*}
f(y_{ij} \mid z_i, {\bf x}_{ij}) = {\rm exp} [\lbrace y_{ij} \eta _{ij} -a(\eta _{ij}) \rbrace \gamma + b(y_i, \gamma )],
\end{eqnarray*}

where *a* and *b* are functions of known form and $\gamma$ is a positive scale factor. In addition, the model postulates that the conditional mean of $Y_{ij} \mid z_i, {\bf x}_{ij}$ relates to a *p*  $\times$ 1 covariate vector ${\bf x}_{ij}$ and random effects $z_i \sim N(0,1)$ through the canonical link function *g* of the generalized linear model of interest. That is, $E(Y_{ij} \mid z_i, {\bf x}_{ij})=g^{-1}(\eta _{ij})$, where


(2) \begin{eqnarray*}
\eta _{ij} = \sigma _u z_i + {\beta {\bf x}_{ij}}
\end{eqnarray*}

is the linear predictor and the *p*-dimensional parameter vector ${ \beta }$ measures the effects of the covariates ${X}$. Note that we decompose the overall random effect in (2) into the standard deviation $\sigma _u$ of the overall random effect and a standardized random effect *z* since we believe that investigators have better intuition about magnitude on the standardized scale than on the linear predictor scale. Investigators have a sense that absolute values of 1 are common and not extreme whereas values above 2 are less common and might be judged extreme and thus frame our problem on this standardized scale to facilitate the definition of extreme as noted by Normand et al. (1997). However, it is straightforward to translate our results to the linear predictor scale.

Inference with generalized linear mixed effects models often focuses on $\beta$ but our objective here is to accurately predict extreme random effects from mixed effects models fitted to clustered data as well as to use predicted random effects, $\tilde{z}$, to identify or “flag” extreme or outlying values such as poorly performing hospitals or geographic areas with high levels of a health outcome. When interest focuses on such extreme random effects, McCulloch and Neuhaus (2021) and McCulloch et al. (2024) showed that predictions obtained by minimizing a weighted mean square error of prediction


(3) \begin{eqnarray*}
\mbox{min}_{\tilde{z}} \mbox{E} [w(z) (\tilde{z} - z)^2],
\end{eqnarray*}

where the weight function $w(z)$ allows for more emphasis to be given to either large or small values of *z*, can provide substantially reduced mean square errors of prediction and appreciably higher correct flagging rates than previously proposed methods for flagging extreme values, while controlling the incorrect flagging rates. McCulloch and Neuhaus (2021) showed that the minimizer of (3) is


(4) \begin{eqnarray*}
\tilde{z}_{w} &=& \mbox{E}[z w(z) \mid {\bf Y}]/\mbox{E}[w(z) \mid {\bf Y}] \\
&=& \frac{\int z w(z) f_{z \mid {\bf Y}}(z \mid {\bf Y}) dz}{\int w(z) f_{z \mid {\bf Y}}(z \mid {\bf Y}) dz} .
\end{eqnarray*}

While McCulloch and Neuhaus (2021) developed results for linear mixed effects models, neither the objective function (3) nor the optimal predictor (4) require that *Y* follow such models so that (4) provides the optimal predictor for any response *Y*, including responses that follow a generalized linear mixed model with a canonical link. When there is no emphasis on extreme random effects, $w(z) \equiv 1$ and the optimal predictor is the standard best predictor


(5) \begin{eqnarray*}
\tilde{z}_{BP} = \mbox{E}[z \mid {\bf Y}].
\end{eqnarray*}

McCulloch and Neuhaus (2021) and McCulloch et al. (2024) proposed 2 useful weight functions which we will employ in this paper: (1) square weighted (SQ), $w(z)=\mbox{exp}(\lambda z^2)$; (2) absolute weighted, $w(z)=\mbox{exp} (\lambda | z|)$ and associated predictors, $\tilde{z}_{SQ}$ and $\tilde{z}_{AB}$, respectively. These weight functions involve a tuning parameter $\lambda$ which controls the magnitude of the emphasis and the value $\lambda =0$ indicates no emphasis on extreme random effects. McCulloch and Neuhaus (2021) showed that with these weight functions one can obtain analytic solutions to the integrals in (4) and closed-form expressions for the optimal predictor when *Y* is Gaussian and follows a linear mixed effects model. However, with non-normal responses from models such as mixed effects logistic, Poisson and gamma, there are not analytic solutions to the integrals in (4) and we must evaluate the integrals using methods such as Gauss-Hermite numerical integration in order to obtain predictions $\tilde{z}_w$.

In order to calculate $\tilde{z}_w$, we first develop some results concerning the conditional density $f(z \mid Y)$ and weighted predictors $\tilde{z}_w$ when *Y* follows a GLMM with a canonical link. Standard calculations show that the conditional distribution of $z_i \mid {\bf Y_i}$ is proportional to


(6) \begin{eqnarray*}
f_{z \mid {\bf Y}}(z_i \mid {\bf Y_i}) & \propto & \prod _j {\rm exp} [\lbrace y_{ij} \eta _{ij} -a(\eta _{ij}) \rbrace \gamma + b(y_{ij}, \gamma )] e^{-z_i^2/2} \\
& \propto & {\rm exp} [\gamma \lbrace Y_{i \cdot } \sigma _u z_i - \Sigma _j a(\eta _{ij}) \rbrace - z_i^2/2]
\end{eqnarray*}

and we see that (6) depends on $\gamma$, $Y_{i \cdot }=\sum _j y_{ij}$, $\sigma _u$ and the parameter $\beta$ of the linear predictor $\eta$. Denote the parameters of the conditional distribution (6) by $\theta =(\gamma , \sigma _u, \beta )$. Since any weighted predictor (4) is based on $f_{z \mid {\bf Y}}(z \mid {\bf Y})$, we see that any weighted predictor is a function of the data only through $Y_{i \cdot }$. That is,


(7) \begin{eqnarray*}
\tilde{z}_{i}=\tilde{z}_i(Y_{i \cdot } \mid \theta , {\bf X}_i, \lambda ).
\end{eqnarray*}

### 2.1 Prediction accuracy

To simplify presentation, we focus on prediction of unusual or extreme random effects in the upper tail, for example, $z>\tau$, and drop the cluster subscript for notational ease, but note that theoretical developments are similar for $z<\tau$ and $|{z}| > \tau$. We assess the prediction accuracy of $\tilde{z}_w$ using the mean square error of prediction conditional on $z>\tau$


(8) \begin{eqnarray*}
&& \mbox{E}[(z-\tilde{z}_w)^2 \mid z> \tau ] \\
&&\quad = \int _{\tau }^{\infty } \int _{{\bf Y}} (z-\tilde{z}_w)^2 f({z, {\bf Y} \mid z>\tau }) d{\bf Y} dz
\end{eqnarray*}

and must evaluate the integrals in (8) using numerical methods. For any canonical link GLMM note that


(9) \begin{eqnarray*}
\mbox{E}[(z-\tilde{z}_w)^2 \mid z>\tau ] = \int \!\!\!\int (z-\tilde{z}_w)^2 dF(Y, z \mid z> \tau )
\end{eqnarray*}

(10) \begin{eqnarray*}
\quad= \int\!\!\! \int _\tau ^\infty [z-\tilde{z}_w(y_\cdot )]^2 f(y_\cdot \mid z)f(z \mid z> \tau )dz d{y_\cdot }
\end{eqnarray*}

Note also that for normally distributed *z*, $f(z \mid z>\tau )=\phi (z)/[1-\Phi (\tau )] I_{z>\tau }$, where $\phi (\cdot )$ and $\Phi (\cdot )$ are the probability density and cumulative distribution functions, respectively, of a standard normal random variable and $I(\cdot )$ is the indicator function. Equation (9) simplifies for discrete responses *Y* as


(11) \begin{eqnarray*}
&&\mbox{E}[(z-\tilde{z}_w)^2 \mid z>\tau ] \\
&&\quad = \sum _y \int _\tau ^\infty (z-\tilde{z}_w)^2 f(y \mid z)f(z \mid z>\tau )dz
\end{eqnarray*}

(12) \begin{eqnarray*}
\qquad &=& \sum _{y_\cdot } \int _\tau ^\infty [z-\tilde{z}_w(y_\cdot )]^2 f(y_\cdot \mid z)f(z \mid z>\tau )dz,
\end{eqnarray*}

where (11) is true for any discrete distribution and (12) is true for canonical link GLMMs.

We can calculate analytic expressions for the distribution of $y_\cdot$ in some important special cases and evaluate the integral in (12) using bounded Gauss–Hermite quadrature (Steen et al., 1969). When there are only cluster-level covariates for the logistic model, the distribution of $y_\cdot$ is binomial with $\mu ={x}^{\prime } \beta$:


(13) \begin{eqnarray*}
f(y_\cdot \mid z) = {n \atopwithdelims ()y_\cdot } \exp \lbrace y_\cdot (\mu +\sigma z) - n \log (1+e^{\mu +\sigma z})\rbrace . \\
\end{eqnarray*}

However, if there are observation level covariates, then $y_\cdot$ is the sum of non-identically distributed Bernoulli variables and follows a Poisson binomial distribution (Hong, 2013). Hong (2013) provides an exact formula with a closed-form expression for the cumulative distribution function of the Poisson binomial distribution which one can use along with bounded Gauss–Hermite quadrature (Steen et al., 1969) to numerically evaluate the integral in (11) in this case. For the Poisson, the distribution is the sum of individual Poisson variables with means $\nu _j=\exp \lbrace x^{\prime }_j\beta +\sigma z\rbrace$ and is therefore Poisson distributed with mean $\nu _\cdot =\sum ^n_{j=1}\exp \lbrace x^{\prime }_j\beta +\sigma z\rbrace .$ For the mixed effects logistic model with no observation level covariates the MSEP conditional on $z>\tau$ is


(14) \begin{eqnarray*}
&& \sum ^n_{k = 0}{n \atopwithdelims ()k} \int _\tau ^\infty [z-\tilde{z}_w(k)]^2 \\
&&\quad\times \,\frac{\exp \lbrace k(\mu +\sigma z) - n \log (1+e^{\mu +\sigma z})\rbrace \phi (z)}{1-\Phi (\tau )}dz. \\
\end{eqnarray*}

For the mixed effects, log link Poisson model the MSEP conditional on $z>\tau$ is


(15) \begin{eqnarray*}
\sum ^\infty _{k = 0}\int _\tau ^\infty [z-\tilde{z}_w(k)]^2 \frac{\exp \lbrace k\log (\nu _\cdot ) - \nu _\cdot \rbrace \phi (z)}{k![1-\Phi (\tau )]}dz.
\end{eqnarray*}

We use bounded Gauss–Hermite quadrature (Steen et al., 1969) to evaluate the integrals in (14) and (15).

### 2.2 Numerical evaluation of mean square error of prediction

We numerically evaluated the mean square error of prediction of each weighted predictor using the expressions in (14) and (15) for binary and Poisson outcomes, respectively. We calculated the difference between the mean square error of prediction of 3 $\tilde{z}_{SQ}$ weighted predictors with $\lambda =.2,.3,.4$ as well as 3 $\tilde{z}_{AB}$ weighted predictors with $\lambda =1.2,1.6, 2.0$ and the mean square error of prediction of the best predictor $\tilde{z}_{BP}$ (5) and produced box plots of the 6 sets of differences. We produced box plots of differences for a range of scenarios using: $\mu =-2, -1$; cluster size=$5, 7, 20, 100$; $\sigma _u$ values ranging from 0.1 to 1.2 by .1 (to 1.0 for Poisson outcomes) and $\tau = 1.28, 1.645, 1.96, 2.33$. The data set of the individual differences displayed in the box plots is available at the Biometrics website on Oxford Academic. One can use these data to obtain the MSEP of any specific predictor and scenario.

**FIGURE 1:**
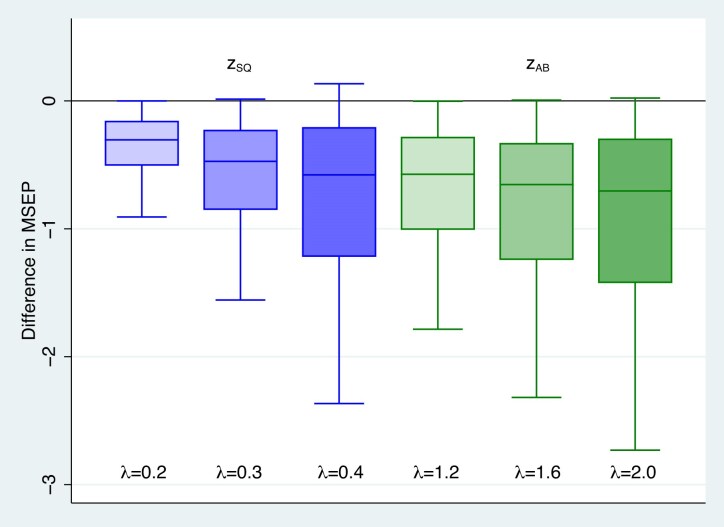
Box plots of differences between the MSEP of $\tilde{z}_{SQ}$ and $\tilde{z}_{AB}$ and the MSEP of $z_{BP}$ for binary outcomes, mixed effects logistic model.

Figure 1 and Supplement Figure 1 of the online supplement present the box plots of MSEP differences for logistic and Poisson models, respectively. Both figures show that the vast majority of the MSEP differences, particularly for $\tilde{z}_{AB}$, are below 0 indicating smaller MSEP for the weighted predictors than $\tilde{z}_{BP}$. In addition, the median differences are all well below 0 indicating that the magnitudes of the reductions of the MSEP for the weighted predictors are often substantial. Overall, the results of our comprehensive numerical evaluation mirror the reductions in MSEP found by McCulloch and Neuhaus (2021) for responses following Gaussian linear mixed effects models; our investigations show that the novel weighted predictors for outcomes from mixed effects logistic and Poisson models have substantially lower mean square error of prediction than the standard best predictor (5).

**FIGURE 2:**
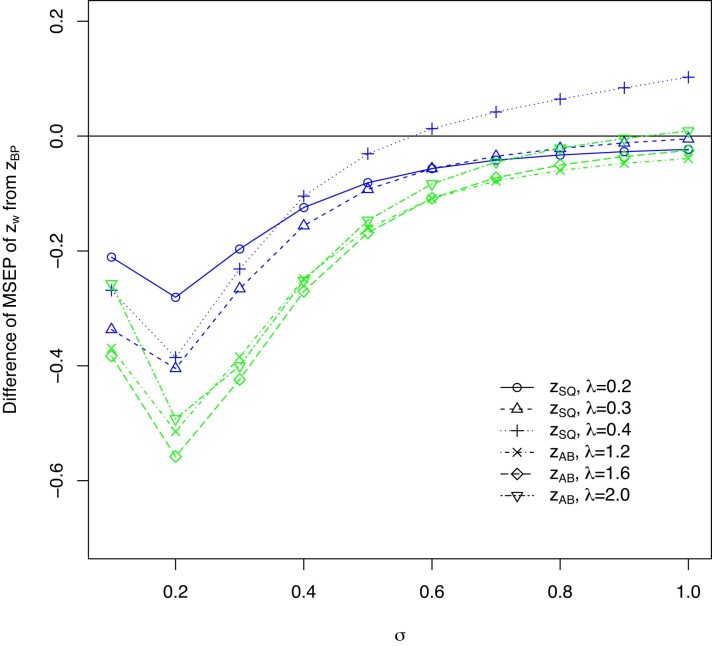
Example with MSEP$(\tilde{z}_{SQ}) >$ MSEP$(\tilde{z}_{BP})$, Poisson MSEP differences for $\tau =1.28$, $n=100$, and $\mu =-2$.

Although the vast majority of the MSEP differences in Figure 1 and Supplement Figure 1 show reduced MSEP of $\tilde{z}_{AB}$ and $\tilde{z}_{SQ}$ compared to $\tilde{z}_{BP}$, there are some scenarios in the Figures, particularly for $\tilde{z}_{SQ}$ with $\lambda =0.4$ where the MSEP$(\tilde{z}_{BP}) <$ MSEP$(\tilde{z}_{SQ})$. Figure 2 presents a specific scenario for Poisson outcomes with $\tau =1.28$, $n=100$ and $\mu =-2$. While Figure 2 shows there are a small percentage of cases where the weighted methods do worse, it also shows that given knowledge of *n*, $\beta$ and $\sigma$, we can choose a value of $\lambda$, for example, $\lambda =0.3$, so that MSEP $(\tilde{z}_{SQ}) <$ MSEP $(\tilde{z}_{BP})$; we can invariably find a more accurate weighted predictor than $\tilde{z}_{BP}$ and it is usually much more accurate.

## 3 FLAGGING

Our second objective is to identify extreme values of the random intercept, *z*. To simplify presentation, we focus on one-sided, upper-tail flagging rules with the goal of identifying extreme values, but we can obtain similar results for lower-tail or two-sided rules. Specifically, we will be concerned with correctly flagging as extreme a random effect *z* that exceeds a pre-determined threshold, $\tau$, on the basis of a predicted value for the random effect, $\tilde{z}$.

While many authors focus on flagging rules that involve a one-sided prediction interval $(\tilde{z}-z_\delta \sigma _{\tilde{z}}, \infty )$ where $\sigma _{\tilde{z}}$ is some measure of accuracy, and $z_\delta$ is the quantile from the upper tail of a standard normal distribution ($1-\Phi (z_\delta )=\delta$), we follow the approach of McCulloch et al. (2024) and focus on flagging rules based on a class of predictors that we call “self-calibrated”. For the case of Gaussian responses *Y* with parameter $\theta$ and for a given class of weighted predictors indexed by $\lambda$, McCulloch et al. (2024) defined a value of the weighting parameter, $\lambda ^{*}(\alpha , \tau , \theta )$, and associated predictor $\tilde{z}_{\lambda ^{*}(\alpha , \tau , \theta )}$ to be “self-calibrated” if it satisfied


(16) \begin{eqnarray*}
\mbox{Pr}\lbrace \tilde{z}_{\lambda ^{*}(\alpha , \tau , \theta )}>\tau \mid z<\tau \rbrace \le \alpha
\end{eqnarray*}

and proposed to flag a cluster as extreme if $\tilde{z}_{\lambda ^{*}(\alpha , \tau , \theta )}>\tau$, a simple rule.

McCulloch et al. (2024) proposed an algorithm to calculate the values of $\lambda ^{*}$ for self-calibrated predictors that took advantage of the closed-form expressions for weighted predictors that exist in the Gaussian response case. Such expressions do not exist for non-Gaussian outcomes and the conditional density $f(z \mid Y)$ is more complicated so that we must develop new algorithms to calculate self-calibrated predictors.

To develop algorithms for non-Gaussian outcomes, we derive some additional results concerning the conditional density $f(z \mid Y)$ and weighted predictors $\tilde{z}_w$ when *Y* follows a GLMM with a canonical link. Recall from (7) that any weighted predictor $\tilde{z}_w$ is a function of the data only through $Y_{\cdot }$. Note that


(17) \begin{eqnarray*}
\frac{\partial f_{z \mid {\bf Y}}(z \mid {\bf Y})}{\partial Y_{\cdot } } = \gamma \sigma _u z f_{z \mid {\bf Y}}(z \mid {\bf Y})
\end{eqnarray*}

and that the derivative of $\tilde{z}_w$ in (4) with respect to $Y_{\cdot }$ is


(18) \begin{eqnarray*}
\frac{\partial \tilde{z}_w}{\partial Y_{\cdot } }= \frac{T_1 T_2 - \gamma \sigma _u T_3^2 }{T_2^2}, \mbox{where}
\end{eqnarray*}

(19) \begin{eqnarray*}
T_1=\int \gamma \sigma _u z^2 w(z) f_{z \mid {\bf Y}}(z \mid {\bf Y}) dz,
\end{eqnarray*}

(20) \begin{eqnarray*}
T_2=\int w(z) f_{z \mid {\bf Y}}(z \mid {\bf Y}) dz, \mbox{and}
\end{eqnarray*}

(21) \begin{eqnarray*}
T_3= \int z w(z) f_{z \mid {\bf Y}}(z \mid {\bf Y}) dz.
\end{eqnarray*}

Note that the denominator of (18), $T_2^2 \ge 0$ and that the numerator is


(22) \begin{eqnarray*}
\gamma \sigma _u \mbox{E}[z^2 w(z) \mid {\bf Y}] \mbox{E}[w(z) \mid {\bf Y}] -\gamma \sigma _u \lbrace \mbox{E}[z w(z) \mid {\bf Y}] \rbrace ^2. \\
\end{eqnarray*}

Consider the random variables $z \sqrt{w(z)}$ and $\sqrt{w(z})$. By the Cauchy–Schwarz inequality


(23) \begin{eqnarray*}
&& \lbrace \mbox{E}[z \sqrt{w(z)} \sqrt{w(z)} \mid {\bf Y}] \rbrace ^2 \\
&&\quad \le \mbox{E}\lbrace [z \sqrt{w(z)} ]^2 \mid {\bf Y} \rbrace \mbox{E}\lbrace [\sqrt{w(z)} ]^2 \mid {\bf Y} \rbrace
\end{eqnarray*}

so that the numerator expression (22) is non-negative and $\tilde{z}_w$ is non-decreasing in $Y_{\cdot }$.

### 3.1 Calculating flagging probabilities

Since $\tilde{z}_w$ is a monotonically increasing function of $Y_{\cdot }$, $\tilde{z}_{\lambda }$ is invertible and we can derive expressions for incorrect and correct flagging probabilities as a function of $Y_{\cdot }$. When flagging $z>\tau$, the incorrect flagging probability is


(24) \begin{eqnarray*}
\mbox{Pr}\lbrace \tilde{z}_\lambda > \tau \mid z<\tau \rbrace &=& \mbox{Pr}\lbrace Y_{\cdot }>\tilde{z}^{-1}_\lambda (\tau ) \mid z<\tau \rbrace\\
&=& \mbox{E}\left[\mbox{Pr}\lbrace Y_{\cdot }>\tilde{z}^{-1}_\lambda (\tau )\rbrace
\mid z, z<\tau \right] \\
&=& \mbox{E}\left[1-F\left(\tilde{z}^{-1}_\lambda (\tau \right) \mid z, z<\tau \right]\\
&=& 1-\int _{-\infty }^{\tau }F\left(\tilde{z}^{-1}_\lambda (\tau )\right)\phi (z)dz/\Phi (\tau ),\\
\end{eqnarray*}

where $F(\cdot )$ is the c.d.f. of $Y_\cdot$ conditional on *z* (and so depends on *z*). We note that the incorrect flagging probability corresponds to 1-specificity in classification.

Similarly, we calculate the correct flagging probability as


(25) \begin{eqnarray*}
&& \mbox{Pr}\lbrace \tilde{z}_\lambda > \tau \mid z > \tau \rbrace\\
&&\quad = \mbox{Pr}\lbrace Y_{\cdot }>\tilde{z}^{-1}_\lambda (\tau ) \mid z>\tau \rbrace \\
&&\quad = \mbox{E}\left[\mbox{Pr}\lbrace Y_{\cdot }>\tilde{z}^{-1}_\lambda (\tau )\rbrace \mid z, z>\tau \right] \\
&&\quad = \mbox{E}\left[1-F\left(\tilde{z}^{-1}_\lambda (\tau )\right) \mid z, z>\tau \right] \\
&&\quad = 1-\int _{\tau }^{\infty }F\left(\tilde{z}^{-1}_\lambda (\tau )\right)\phi (z)dz/(1-\Phi (\tau )),
\end{eqnarray*}

where $F(\cdot )$ is the c.d.f. of $Y_\cdot$ conditional on $z.$ We note that a correct flagging rate corresponds to sensitivity in classification.

We cannot analytically evaluate the integrals in (24) and (25) but we can write them in the form


(26) \begin{eqnarray*}
\int _{-\infty }^{\tau }f(z)\phi (z)dz = \int _{-\infty }^{\tau } f(z)\frac{\exp \lbrace -z^2/2\rbrace }{\sqrt{2\pi }}dz,
\end{eqnarray*}

which we evaluate using bounded Gauss–Hermite quadrature (Steen et al., 1969). Section 2.1 provides expressions for the c.d.f. of $Y_{\cdot }$, $F(\cdot )$, for logistic models with no individual-level covariates and Poisson models.

To evaluate the integrals in (24) and (25), we need to determine $\tilde{z}^{-1}_\lambda (\tau )$. To do so, we use the algorithm below. Recall that given inputs $\lambda$, $\mu$, $\sigma _u$, and $y_{\cdot }$ and cluster size, we can calculate any weighted predictor, including $\tilde{z}_{SQ}$ and $\tilde{z}_{AB}$, using (4) and Gauss–Hermite quadrature.

**Algorithm**

1. For discrete outcomes such as those from logistic and Poisson GLMMs, using inputs $\lambda$, $\mu$, $\sigma _u$, and cluster size solve for the value of $y_{\cdot }$ that gives $\tilde{z}_{w}(y_{\cdot }) < \tau < \tilde{z}_{w}(y_{\cdot } + 1)$ using an exhaustive search and call it $y_{\cdot , \tau }$. For continuous outcomes such as Gamma random variables we numerically solve $\tilde{z}_{w}(y_{\cdot }) = \tau$ for $y_{\cdot , \tau }$.
2. This solves for $\tilde{z}^{-1}_\lambda (\tau )$ in (24) or (25) since $y_{\cdot , \tau } < \tilde{z}^{-1}_\lambda (\tau )$.
3. Evaluate (24) or (25) at $y_{\cdot , \tau }$.

### 3.2 Algorithms for self-calibration

To self-calibrate a weighted predictor we need to solve for the value $\lambda ^{*}$ that satisfies the inequality (16). To do so, we use the algorithm above and a bisection algorithm or another root finding algorithm to solve for $\lambda ^{*}$ that gives the desired incorrect flagging rate. Alternatively, we could use a simulation-based approach and the algorithm below.

1. For given values of $\gamma , \mu , \sigma _u, \beta , {\bf X}$ and *F*, calculate the distribution of $Y_{\cdot }$ conditional on $z < \tau$ numerically or by simulation.
2. For a given $\alpha$ read off the upper quantile, $y_{\alpha }$, as the smallest value satisfying:
  \begin{eqnarray*}
\mbox{Pr}\lbrace Y_{\cdot } > y_{\alpha } \mid z< \tau \rbrace \le \alpha .
\end{eqnarray*}
3. To be self-calibrated, we want $\mbox{Pr}\lbrace \tilde{z}_{\lambda }>\tau \mid z< \tau \rbrace \le \alpha .$ Since
  \begin{eqnarray*}
\mbox{Pr}\lbrace \tilde{z}_{\lambda }>\tau \mid z< \tau \rbrace = \mbox{Pr}\lbrace Y_{\cdot } >\tilde{z}_{\lambda } ^{-1} (\tau ) \mid z< \tau \rbrace ,
\end{eqnarray*}
  we set $\tilde{z}_{\lambda } ^{-1} (\tau ) = y_{\alpha }$ or equivalently, we can use a root finding algorithm to solve for $\lambda$ in
  (27) \begin{eqnarray*}
\tilde{z}_{\lambda }(y_{\alpha })=\tau .
\end{eqnarray*}

### 3.3 Equivalence of self-calibrated predictors

If we can solve (27), then the correct and incorrect flagging probabilities will only be a function of the probabilities of $Y_{\cdot } \ge y_{\alpha }$ and all flagging rules will be equivalent (though the predictions will be different). In particular, suppose there are 2 predictors ($k=1,2$), we can self-calibrate by solving $\tilde{z}_{k,\lambda _k}(y_\alpha )=\tau .$ Then


(28) \begin{eqnarray*}
\tilde{z}_{1,\lambda _1}(y_\alpha )&\ge& \tau \mbox{ iff } Y_\cdot \ge \tilde{z}^{-1}_{1, \lambda _1}(\tau )=y_\alpha \mbox{ iff } Y_\cdot \ge y_\alpha \\
&=&\tilde{z}^{-1}_{2, \lambda _2}(\tau ) \mbox{ iff } \tilde{z}_{2,\lambda _2}(y_\alpha )\ge \tau .
\end{eqnarray*}

That is *all flagging rules will be equivalent*.

## 4 CALCULATIONS TO EVALUATE FLAGGING PERFORMANCE

### 4.1 Numerical evaluation of the incorrect flagging rate

For both binary and Poisson outcomes, we calculated incorrect flagging rates by evaluating (24) using numerical integration and evaluated the performance of self-calibrated predictors, as well as $\tilde{z}_{BP}$. Our flagging rule for $\tilde{z}_{BP}$ was to flag a cluster if $\tilde{z}_{BP} > \tau$, that is, a flagging rule with no prediction interval. Below, we show that even this simple flagging rule is very conservative. Adding a prediction interval will only increase the conservatism of the rule. We calculated $\tilde{z}_{BP}$ as well as the self-calibrated versions of $\tilde{z}_{SQ}$ and $\tilde{z}_{AB}$ using (4) and the algorithm in Section 3.2 for a finely spaced grid of values of $\sigma _u$ and specified values of cluster size *n* for the predictor whose incorrect flagging rate was closest to the nominal $\alpha$. For each predictor, we calculated the incorrect flagging rate for a range of scenarios using: $\mu =-1, -0.5, 0, 0.5, 1$; cluster size $=5, 10, 20, 100$; $\tau =1.28, 1.645, 1.96, 2.33$; and $\alpha =.05,.1$. We produced box plots of the incorrect flagging rates separately for the 2 $\alpha$ values.

For 295 scenarios (14%) with binary responses, the incorrect flagging rate of $\tilde{z}_{BP}$ was greater than the nominal $\alpha$ due to the highly discrete distribution of $Y_\cdot$ which restricts the distribution of achievable incorrect flagging rates. Since weighted predictors shrink less than $\tilde{z}_{BP}$ it is not feasible to obtain self-calibrated predictors for these scenarios and the plots we present do not include these infeasible scenarios. With Poisson responses, the distributions of $Y_\cdot$ and achievable incorrect flagging rates have more support points than with binary responses and infeasible scenarios where the incorrect flagging rate of $\tilde{z}_{BP}$ was greater than the nominal $\alpha$ were much rarer, occurring only once. As with the MSEs of Section 2.2 the resulting incorrect flagging rates are available at the Biometric website on Oxford Academic and one can examine specific scenarios using the data in the Supplementary Materials.

**FIGURE 3:**
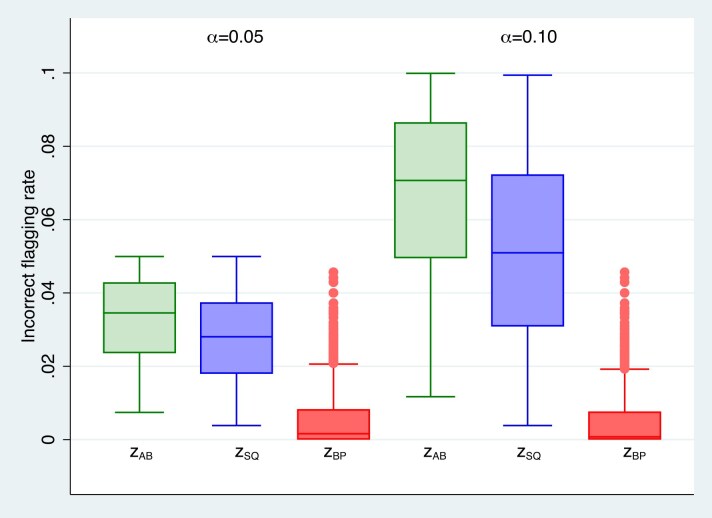
Box plots of incorrect flagging rates for $\tilde{z}_{AB}$, $\tilde{z}_{SQ}$, and $\tilde{z}_{BP}$ with binary outcomes.

**FIGURE 4:**
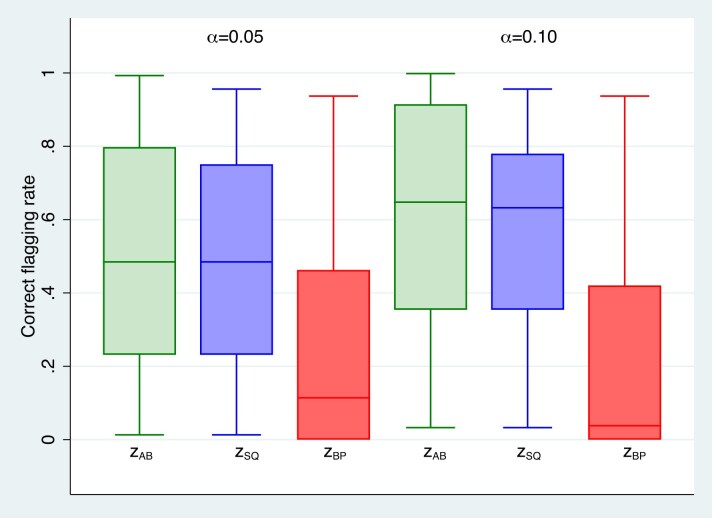
Box plots of correct flagging rates for $\tilde{z}_{AB}$, $\tilde{z}_{SQ}$, and $\tilde{z}_{BP}$ with binary outcomes.

Figure 3 and Supplement Figure 2 of the online supplement present box plots of the incorrect flagging rates for logistic and Poisson models, respectively, based on all of the feasible scenarios. Figure 3 for the logistic model shows that the incorrect flagging rates for $\tilde{z}_{BP}$ are far below nominal with 75th percentiles less than 0.01 for both $\alpha =0.05$ and $\alpha =0.10$ indicating overly conservative flagging. The incorrect flagging rates for both $\tilde{z}_{AB}$ and $\tilde{z}_{SQ}$ are much closer to nominal, although still conservative, with median values of 0.03 for $\tilde{z}_{AB}$ and 0.03 for $\tilde{z}_{SQ}$ for $\alpha =0.05$ and median values of 0.07 for $\tilde{z}_{AB}$ and 0.05 for $\tilde{z}_{SQ}$ for $\alpha =0.10$. The discreteness of the distribution of $Y_{\cdot }$ prevents us from constructing self-calibrated weighted predictors with incorrect flagging rates closer to nominal. More scenarios were feasible for $\alpha =0.10$ than for $\alpha =0.05$ so that the $\tilde{z}_{BP}$ values displayed in Figure 3 (and Figure 4) differed slightly between the 2 different $\alpha$ although $\tilde{z}_{BP}$ does not depend on $\alpha$. The findings in the online supplement for the Poisson model mirror those of the logistic model in Figure 3; the incorrect flagging rates for $\tilde{z}_{BP}$ are far below nominal indicating overly conservative flagging and the incorrect flagging rates for both $\tilde{z}_{AB}$ and $\tilde{z}_{SQ}$ are much closer to nominal.

### 4.2 Numerical evaluation of the correct flagging rate

We followed the numerical integration approach of Section 4.1 using the same range of scenarios to evaluate correct flagging rates of predictors $\tilde{z}_{BP}$, $\tilde{z}_{AB}$, and $\tilde{z}_{SQ}$. As in Section 4.1, Figure 4 and Supplement Figure 3 of the online supplement present box plots of correct flagging rates for logistic and Poisson models, respectively, separately for $\alpha =0.05$ and $\alpha =0.10$. Figure 4 for the logistic model shows that the correct flagging rates for $\tilde{z}_{BP}$ are consistently and substantially lower than the rates for $\tilde{z}_{AB}$ and $\tilde{z}_{SQ}$; the median correct flagging rate for $\tilde{z}_{BP}$ is 0.07, while the median correct flagging rates for $\tilde{z}_{AB}$ and $\tilde{z}_{SQ}$ are both 0.57. The findings in the online supplement for the Poisson model mirror those of the logistic model in Figure 4; the correct flagging rates for $\tilde{z}_{BP}$ are consistently and substantially lower than the rates for $\tilde{z}_{AB}$ and $\tilde{z}_{SQ}$.

## 5 ASTHMA EXAMPLE

We illustrate our results through the analysis of data from a study of asthma-related ED visits among children and adolescents (Bardach et al., 2022). Using data from claims databases, Bardach et al. (2022) identified asthma-related ED visits for patients ages 3–21. The outcome of interest was a binary indicator of whether or not a subject had an asthma-related ED revisit after the initial ED visit and the data were clustered within zip codes. The data set includes 2 covariates: CES4.0 pollution score (California Office of Environmental Health Hazard Assessment, 2023) and zip code average income (California Franchise Tax Board, 2024) which we standardized to have 0 means and unit variances using sample estimates. The objective of Bardach et al. (2022) was to assess whether there was an association between a subject having an early visit (within 14 days of the initial ED visit) with a primary care provider or an asthma specialist with the probability of an asthma-related ED revisit (between 2 weeks and 1 year after the initial visit). The objective of our secondary analysis is to predict and flag zip codes with high ED readmission rates for asthma, adjusting for pollution score and average income. For our analyses, we randomly partitioned the data within zip codes into training and validation samples. We used the predictions from the training sample to predict and flag the zip code-specific ED readmission rates for the validation sample. We restricted our analysis to zip codes with $n_i \ge 100$ for more accurate estimated proportions $\hat{p}_i$ in validation sample. Otherwise, we are less sure that the zip codes identified as high using the validation data are truly high. This restriction left us with $m=248$ zip codes and *n* = 44 678 participants.

We fit a mixed-effects logistic model with a random intercept and the 2 zip code level covariates to the training sample data to obtain estimates $\hat{\mu }$, $\hat{\beta }_{CES4.0}$, $\hat{\beta }_{income}$, and $\hat{\sigma }_{u}$ which we used to calculate “true” $z_i$ values for validation sample as


**FIGURE 5:**
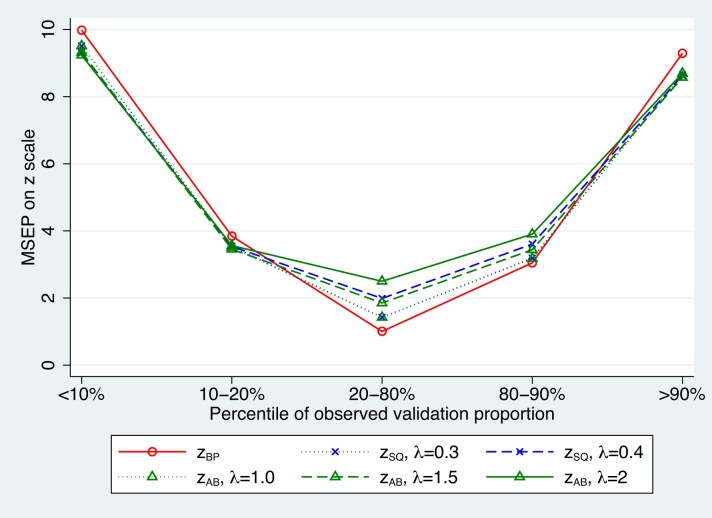
Estimated mean square error of prediction in asthma validation data by ordered subgroups (percentiles). Large $(n > 100)$ clusters only.

\begin{eqnarray*}
\hat{z}_{i, Valid} = [\mbox{logit} (\hat{p}_{i, Valid}) - \hat{\eta _{i}}]/\hat{\sigma }_{u},
\end{eqnarray*}

where $\hat{p}_{i, Valid}$ is the observed rate of asthma-related ED revisits for the $i^{th}$ zip code and $\hat{\eta _{i}}=\hat{\mu } + \hat{\beta }_{CES4.0} \mbox{CES4.0}_i + \hat{\beta }_{income} \mbox{income}_i$. The online supplement presents the results of additional analyses of the asthma data using a model that includes no covariates to illustrate the effects of covariate adjustment. We assessed the prediction accuracy of predictors $\tilde{z}_{BP}$, $\tilde{z}_{SQ} (\lambda =.3,.4)$, and $\tilde{z}_{AB} (\lambda =1.0, 1.4,1.8)$ by calculating estimated MSEPs, the average of $(\tilde{z}_{i, Test} - \hat{z}_{i, Valid})^2,$ by ordered subgroups of $\hat{z}_{i, Valid}$ and present the results in Figure 5. Figure 5 shows that in the 2 extreme subgroups (the smallest and largest 10% of the clusters according to the validation data), the MSEPs of the weighted predictors are all smaller than the MSEP of the best predictor, $\tilde{z}_{BP}$, indicating more accurate prediction of extreme random effects.

To assess flagging with the asthma data we designate the zip codes with top 10% of $\hat{p}_{i,Valid}$ as “extreme” $(m=25)$ and the remaining 90% $(m=223)$ as not-“extreme”. Our objective is to flag zip codes with $z > 1.28$, that is, top 10% of readmission random effects and we allow an incorrect flagging rate of $\alpha =0.1$. We calculated $\tilde{z}_{BP}$ and the self-calibrated $\tilde{z}_{SQ}$, $\tilde{z}_{AB}$ using the training sample and the simulation-based algorithms of Sections 2 and 3 and flagged zip codes with $\tilde{z} > \tau =1.28$. We compared the flagged training sample zip codes to the validation sample “extreme” zip codes and present the results in Table 1. $\tilde{z}_{BP}$ is highly conservative and incorrectly flagged only 1 of the 223 non-“extreme” zip codes for a rate of 0.004, far below the nominal rate of 0.1. Also, $\tilde{z}_{BP}$ correctly flagged only 1 of the 25 “extreme” zip codes. Both $\tilde{z}_{SQ}$ and $\tilde{z}_{AB}$ incorrectly flagged 21 of the 223 non-“extreme” zip codes for a rate of 0.094, close to nominal incorrect flagging rate of 0.1. Also, both $\tilde{z}_{SQ}$ and $\tilde{z}_{AB}$ correctly flagged 9 of the 25 “extreme” zip codes, many more than $\tilde{z}_{BP}$. We were able to self-calibrate both $\tilde{z}_{SQ}$ and $\tilde{z}_{AB}$ so that the 2 flagging rules based on these 2 predictors flagged exactly the same zip codes, consistent with the findings of Section 3.3. While the low correct flagging rates may seem surprising, the online Supplement presents the results of an oracle method that only flagged an average of 8.5 (simulation SE 0.04) of the 25 extreme zip codes.

**TABLE 1:** Agreement between zip codes flagged as extreme (flag) using training data and whether they were in the top 10% (extreme) of the validation data for various predictors and flagging rules using asthma data.

Flagging rule	Flag/extreme	$\overline{\mbox{flag}}/{\mbox{extreme}}$	Flag/$\overline{\mbox{extreme}}$	$\overline{\mbox{flag}}/ \overline{\mbox{extreme}}$
$\tilde{z}_{BP}$	1	24	1	222
Self-calibrated $\tilde{z}_{SQ}$	9	16	21	202
Self-calibrated $\tilde{z}_{AB}$	9	16	21	202

The overbar denotes not. Rules flag a zip code if $\tilde{z} > 1.28$.

## 6 DISCUSSION AND RECOMMENDATIONS

In this paper, we extend the weighted prediction, as well as flagging, methods, and results of McCulloch and Neuhaus (2021) and McCulloch et al. (2024) for Gaussian responses to settings with commonly occurring non-Gaussian responses such as binary and Poisson. These extensions required the heavy use of numerical methods such as bounded Gauss–Hermite quadrature, along with the development of novel algorithms and supporting theory since analogs of the closed-form expressions McCulloch and Neuhaus (2021) and McCulloch et al. (2024) used for Gaussian responses do not exist for non-Gaussian outcomes. Our extensive numerical results for binary and Poisson outcomes, as well as the analysis of binary outcomes from the asthma readmission study (Bardach et al., 2022) showed that when interest focuses on prediction of extreme random effects for non-Gaussian outcomes, the weighted prediction methods consistently outperformed standard prediction methods based on overall best prediction. In particular, the absolute weighted predictor, $\tilde{z}_{AB}$, often reduced the mean square error of prediction of standard best prediction methods by more than 50%. As well as providing more accurate prediction of extreme random effects, the weighted prediction methods provided much better control of incorrect flagging rates and substantially greater correct flagging rates than standard best prediction methods. In particular, the incorrect flagging rates of the absolute weighted predictor, $\tilde{z}_{AB}$, were consistently close to nominal while the incorrect flagging rates of standard best prediction methods were consistently far below nominal, leading to very low correct flagging rates. The correct flagging rates of $\tilde{z}_{AB}$ were often 50% larger than those of standard best prediction methods.

While much of the paper focused on responses from canonical link GLMMs, the expressions for the optimal weighed predictor (4), incorrect (24) and correct (25) flagging probabilities hold in general and one could evaluate the relevant integrals using Monte Carlo numerical methods instead of the the quadrature methods we focus on in this paper. Similarly, one could use Monte Carlo methods to evaluate the multivariate distribution of ${\bf Y_i}$ and include this distribution in a modified algorithm to calculate self-calibrated predictors.

Finally, when the objective is random effects prediction, we believe that investigators know what extreme random effects are, that is, what the threshold $\tau$ is, as well as know how much more heavily to weight contributions from extreme random effects and that this knowledge guides the choice of $\lambda$. However, assuming known values of $\beta$, $\sigma$ and cluster size, an investigator could also choose $\lambda$ as the value that minimizes an expression for the mean square error of prediction such as Equation (12) or chose $\lambda$ to balance performance across a range of values of $\tau , \beta ,$ and $\sigma .$

## Supplementary Material

ujaf094_Supplemental_Files Web Appendices, Tables, and Figures referenced in Sections 2.2, 4.1, 4.2, and 5, as well as data and code to implement the algorithms in Section 3 are available with this paper at the Biometrics website on Oxford Academic.

## Data Availability

Our data use agreement does not allow us to share the California Department of Public Health data. However, we created a simulated dataset that: (1) includes the publically available covariates, (2) shares the same structural characteristics as the actual data, and (3) is in exactly the same format as the actual data. These data and the results for the simulated data are available with this paper at the Biometrics website on Oxford Academic.
