# Supplementary material for: Improved prediction and flagging of extreme random effects for non-Gaussian outcomes using weighted methods
Source: Biometrics. 2025 Jul 26;81(3):ujaf094. doi: 10.1093/biomtc/ujaf094 (PMC12309285; doi:10.1093/biomtc/ujaf094)
Supplement: ujaf094_Supplemental_Files — Web Appendices, Tables, and Figures referenced in Sections 2.2, 4.1, 4.2, and 5, as well as data and code to implement the algorithms in Section 3 are available with this paper at the Biometrics website on Oxford Academic. [file ujaf094_supplemental_files.zip › BIOM2024617M.supplement.pdf]

Supplementary Materials for Improved prediction and flagging of extreme random effects for non-Gaussian outcomes using weighted methods by John M. Neuhaus, Charles E. McCulloch, and Ross Boylan

This Appendix provides supplementary results from the numerical evaluation of mean square error of prediction, incorrect and correct flagging rates for Poisson outcomes related to "Improved prediction and flagging of extreme random effects for non-Gaussian outcomes using weighted methods". Specifically, for outcomes following mixed effects Poisson models this Appendix provides for :1) box plots of the differences between the mean square error of prediction of three $\tilde{z}_{SQ}$ weighted predictors with $\lambda = .2, .3, .4$ as well as three $\tilde{z}_{AB}$ weighted predictors with $\lambda = 1.2, 1.6, 2.0$ using Equation (14) of manuscript and the mean square error of prediction of the best predictor $\tilde{z}_{BP}$; 2) box plots of the incorrect flagging rates of self-calibrated predictors $\tilde{z}_{SQ}$ and $\tilde{z}_{AB}$, as well as $\tilde{z}_{BP}$ obtained by evaluating Equation (24) of manuscript using numerical integration; and 3) box plots of the correct flagging rates of self-calibrated predictors $\tilde{z}_{SQ}$ and $\tilde{z}_{AB}$, as well as $\tilde{z}_{BP}$ obtained by evaluating Equation (25) of manuscript using numerical integration. In addition, this Appendix includes a section with the results of additional analyses of the asthma data using a model that includes no covariates in order to illustrate the effects of covariate adjustment and a section with the results of an "oracle" prediction method which provides an upper bound on performance.

1 Mean square errors of prediction for Poisson outcomes

We produced box plots of differences in mean square errors of prediction for a range of scenarios using: $\mu = -2, -1$; cluster size=$5, 7, 20, 100$; σ_u values ranging from 0.1 to 1.0 by .1 and $\tau = 1.28, 1.645, 1.96, 2.33$.

We calculated the incorrect and correct flagging rates for a range of scenarios

using: $\mu = -1, -0.5, 0, 0.5, 1$; cluster size $= 5, 10, 20, 100$; $\tau = 1.28, 1.645, 1.96, 2.33$; and $\alpha = .05, .1$.

Supplement Figure 1 shows that the vast majority of the MSEP differences for Poisson outcomes, particularly for $\tilde{z}_{AB}$, are below zero indicating smaller MSEP for the weighted predictors than $\tilde{z}_{BP}$. In addition, the median differences are all well below zero indicating that the magnitudes of the reductions of the MSEP for the weighted predictors are often substantial. Overall, the results for Poisson outcomes in Supplement Figure 1 mirror the reductions in MSEP we displayed for binary outcomes in the main manuscript.

Supplement Figure 2 shows that the incorrect flagging rates for $\tilde{z}_{BP}$ are far below nominal with 75th percentiles less than 0.01 for both $\alpha = 0.05$ and $\alpha = 0.10$ indicating overly conservative flagging. The incorrect flagging rates for both $\tilde{z}_{AB}$ and $\tilde{z}_{SQ}$ are much closer to nominal, although still somewhat conservative with median values of 0.03 for $\tilde{z}_{AB}$ and 0.02 for $\tilde{z}_{SQ}$ for $\alpha = 0.05$ and median values of 0.05 for $\tilde{z}_{AB}$ and 0.02 for $\tilde{z}_{SQ}$ for $\alpha = 0.10$. The discreteness of the distribution of $Y_{\cdot}$ prevents us from constructing self-calibrated weighted predictors with incorrect flagging rates closer to nominal.

Supplement Figure 3 shows that the correct flagging rates for $\tilde{z}_{BP}$ are consistently and substantially lower than the rates for $\tilde{z}_{AB}$ and $\tilde{z}_{SQ}$; the median correct flagging rate for $\tilde{z}_{BP}$ is 0.50 while the median correct flagging rates for $\tilde{z}_{AB}$ and $\tilde{z}_{SQ}$ are 0.87 and 0.69, respectively. Overall, the incorrect and correct flagging rates for Poisson outcomes in Supplement Figures 2 and 3 mirror the improved flagging performance of weighted predictors that we displayed for binary outcomes in the main manuscript; the weighted predictors provided much higher correct flagging rates while controlling the incorrect flagging rate.

2 Effects of covariate adjustment with asthma data

We present the results of additional analyses of the asthma data using a model that includes no covariates in order to illustrate the effects of covariate adjustment. As in Section 5, we restricted our analysis to zip codes with $n_i \geq 100$ for more accurate estimated proportions $\hat{p}_i$ in validation sample.

In the additional analyses we fit a mixed-effects logistic model with a random intercept and no covariates to the training sample data to obtain estimates $\hat{\mu}$ and $\hat{\sigma}_u$ which we used to calculate predictions $\tilde{z}_{i,Train}$ for the training sample and "true" z_i values for validation sample as

$$\hat{z}_{i,Valid} = [\text{logit}(\hat{p}_{i,Valid}) - \hat{\mu}]/\hat{\sigma}_u.$$

We designated the zip codes with top 10% of $\hat{z}_{i,Valid}$ as "extreme" ($m = 25$), the remaining 90% ($m = 223$) as not-"extreme" and allowed an incorrect flagging rate of $\alpha = 0.1$.

We calculated $\tilde{z}_{BP}$ and the self-calibrated $\tilde{z}_{SQ}$, $\tilde{z}_{AB}$ using the training sample and the simulation-based algorithms of Sections 2 and 3 and flagged zip codes with $\tilde{z} > \tau = 1.28$. As in the analysis including the two covariates, we were able to self-calibrate both $\tilde{z}_{SQ}$ and $\tilde{z}_{AB}$ with no covariates so that the two flagging rules based on these two predictors flagged exactly the same zip codes, consistent with the findings of Section 3.3. 216 zip codes are not flagged by either the model with covariates or the model without covariates and 28 zip codes are flagged by both models. Two zip codes each were flagged by one model but not the other. The income measure had a modest negative association with readmission and the flagging discrepancies between the two models occurred with zip codes that had average incomes far from the mean. Adjusting for covariates in these cases pushed the predicted random effects below and

above the cutoff τ. However, these cases were very infrequent with the asthma data and the flagging performance based on the two models was nearly identical.

3 Oracle method for asthma example

Since we do not know if the zip codes we designated as extreme actually correspond to zip codes with extreme values of z, we also assessed an "oracle" prediction method as an upper bound on performance. In this analysis we simulated random effects and asthma readmission data from a logistic mixed model with no covariates using the values of the estimated parameters ($\mu = -0.822$ and $\sigma = 0.149$) and the observed sample sizes for each zip code from the validation dataset. Excluding covariates simplified both the simulation and flagging analysis but provided relevant oracle results. For each simulated dataset we designated the top 10% ($n = 25$) zip codes with the largest observed proportions as "extreme." We also know which zip codes have values of the random effects, z, that exceed the threshold of $\tau = 1.28$; those form our "oracle" flagging rule. For each simulated dataset we recorded the proportion of the oracle-flagged zip codes that were actually designated as extreme. We repeated our simulation about 2,500 times. On average, the oracle method only flagged 8.5 (simulation SE 0.04) of the 25 extreme zip codes. This low flagging rate may seem surprising, but because of the low value of σ (and resulting poor separation of the clusters) the correlation between the observed proportion in the validation sample and the true value of z_i is low – only about 0.5. This low "signal" is not unusual in many flagging contexts where cluster to cluster variation and intraclass correlations are low. In this light, our flagging rules based on $\tilde{z}_{AB}$ and $\tilde{z}_{SQ}$ performed well since they flagged 8 clusters each.

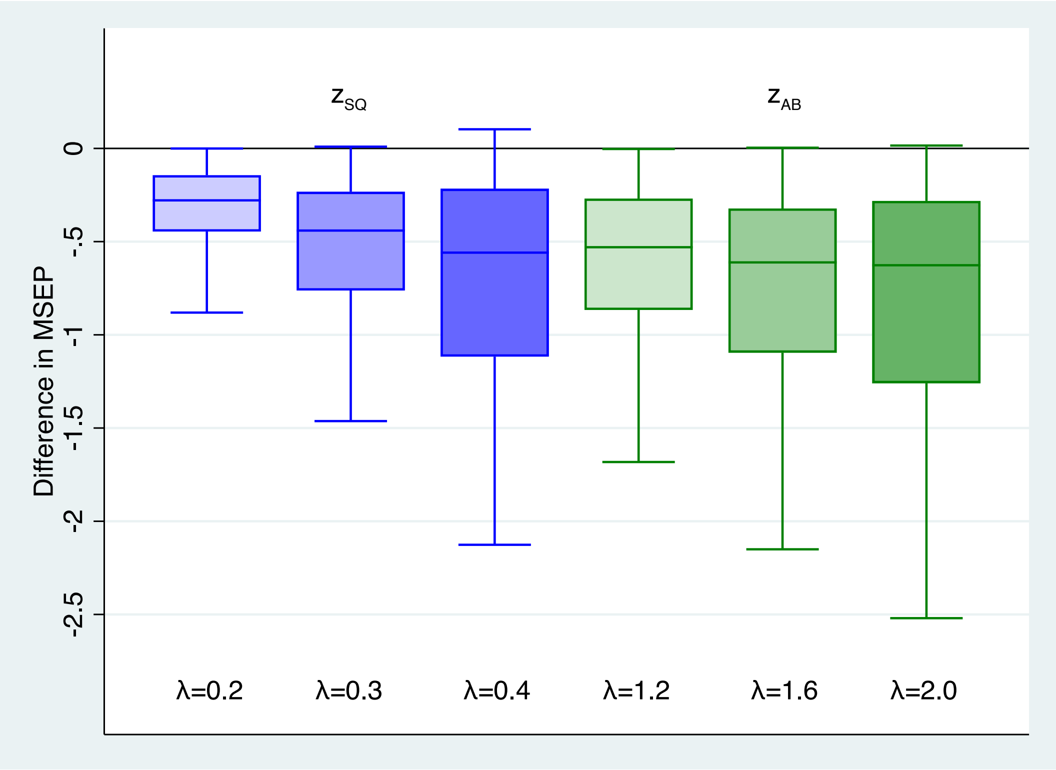

Figure 1: Box plots of differences between the MSEP of $\tilde{z}_{SQ}$ and $\tilde{z}_{AB}$ and the MSEP of $\tilde{z}_{BP}$ for Poisson outcomes, mixed effects Poisson model.

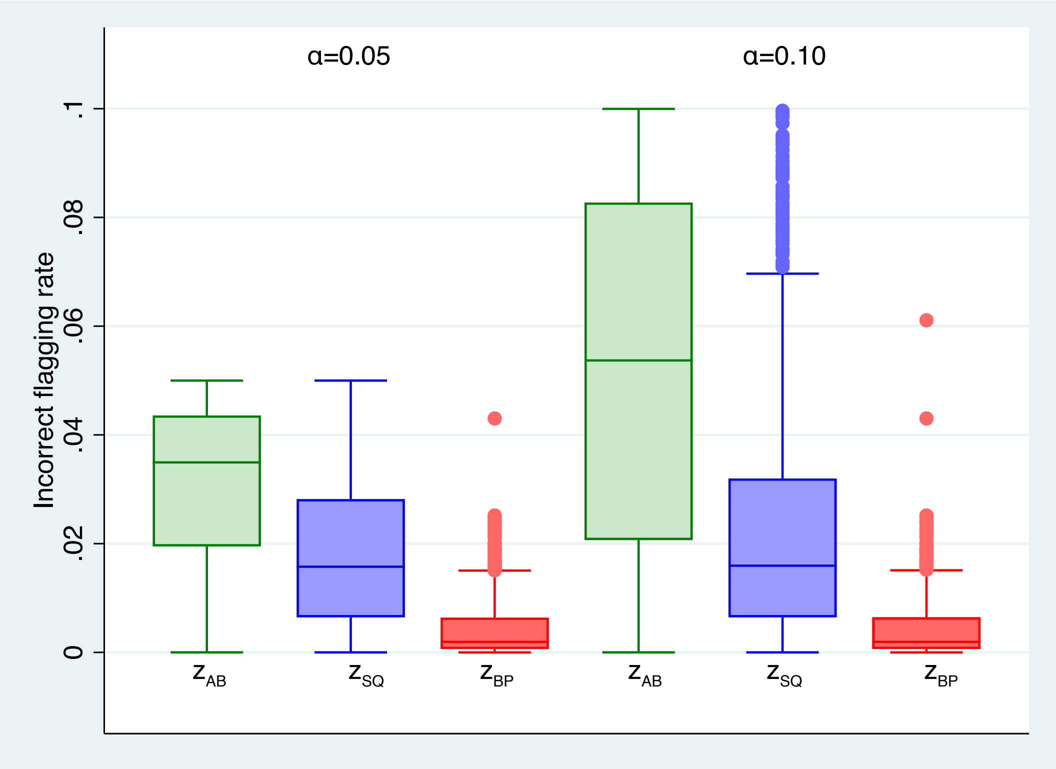

Figure 2: Box plots of incorrect flagging rates for $\tilde{z}_{AB}$, $\tilde{z}_{SQ}$ and $\tilde{z}_{BP}$ with Poisson outcomes.

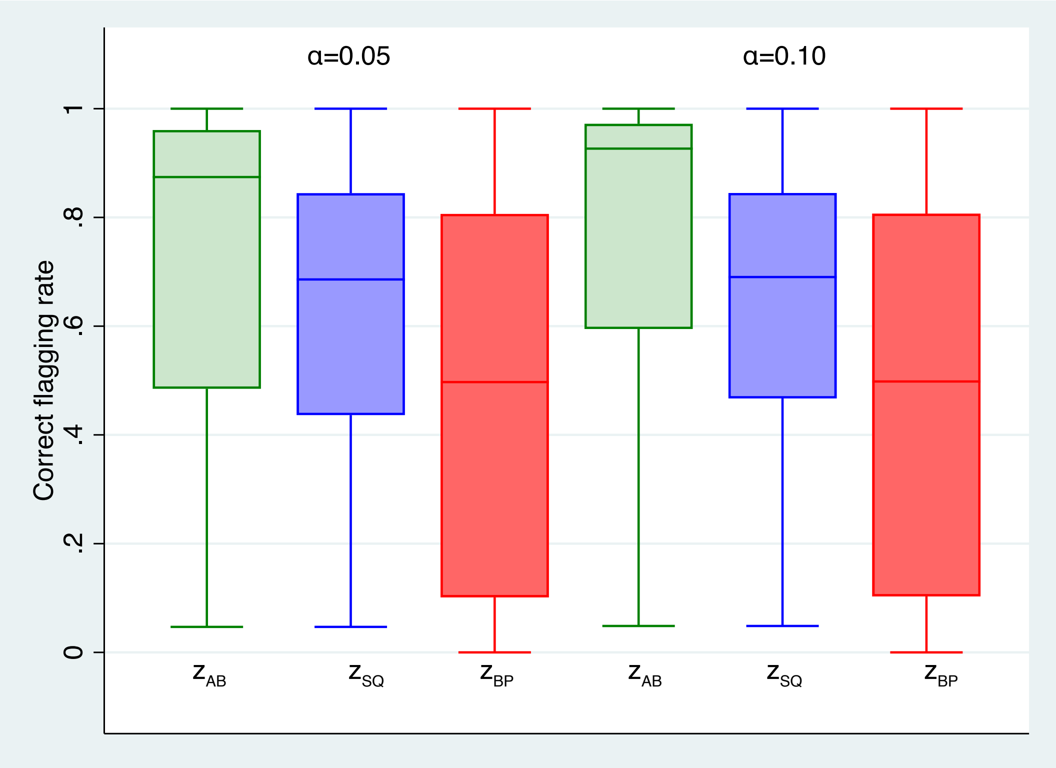

Figure 3: Box plots of correct flagging rates for $\tilde{z}_{AB}$, $\tilde{z}_{SQ}$ and $\tilde{z}_{BP}$ with Poisson outcomes.
