# Supplementary material for: Improved prediction and flagging of extreme random effects for non-Gaussian outcomes using weighted methods
Source: Biometrics. 2025 Jul 26;81(3):ujaf094. doi: 10.1093/biomtc/ujaf094 (PMC12309285; doi:10.1093/biomtc/ujaf094)
Supplement: ujaf094_Supplemental_Files — Web Appendices, Tables, and Figures referenced in Sections 2.2, 4.1, 4.2, and 5, as well as data and code to implement the algorithms in Section 3 are available with this paper at the Biometrics website on Oxford Academic. [file ujaf094_supplemental_files.zip › Supplemental_data_asthma_example/README file.docx]

README file for Improved prediction and flagging of extreme random effects for non-Gaussian

outcomes using weighted methods: Asthma example

Our data use agreement does not allow us to share the data, which comes from the California Department of Public Health. However, we created a simulated dataset that: a) includes the publicly available covariates, b) shares the same structural characteristics as the actual data, and c) is in exactly the same format as the actual data. We have included the code we used in the manuscript as well as the results for the simulated data, which are similar to those reported in the manuscript.

The supplementary material for the asthma example is a Stata .do file that requires two data inputs: the asthma data (“asthma plus income plus CES data modified v2.csv”) and weights and abscissae for bounded Gauss-Hermite quadrature (“bounded GH quadrature points.xlsx”). It creates some of the plots displayed in the manuscript, and the results used for flagging and whether or not the zip code was flagged. Those results are stored as both a Stata data file and a .csv file with the names “flagging plot big modified results.dta” and “flagging plot big modified results.csv”. Those output datasets contain:

n_est_365_14 = number of asthma cases in a zip code in the training sample

n_val_365_14 = number of asthma cases in a zip code in the validation sample

y_est_365_14 = number of readmissions in a zip code in the training sample

y_val_365_14 = number of readmissions in a zip code in the validation sample

std_avg_income = standardized average income

std_ces40 = standardized pollution burden score

zipcode_masked = masked zip code identifier

xb_noc = fitted linear predictor with no covariates (noc)

xb_cov = fitted linear predictor with covariates (cov)

lam_AB_cov = self-calibrated value of weighting parameter for z_AB with covariates

lam_SQ_cov = self-calibrated value of weighting parameter for z_SQ with covariates

z_AB_cov = weighted predictor z_AB using lambda equal to lam_AB_cov

z_SQ_cov = weighted predictor z_SQ using lambda equal to lam_SQ_cov

z_BP_cov = usual best predictor with covariates

lam_AB_noc = self-calibrated value of weighting parameter for z_AB with no covariates

lam_SQ_noc = self-calibrated value of weighting parameter for z_SQ with no covariates

z_AB_noc = weighted predictor z_AB using lambda equal to lam_AB_noc

z_SQ_noc = weighted predictor z_SQ using lambda equal to lam_SQ_noc

z_BP_noc = usual best predictor with no covariates

flag_AB_noc = 1 if a cluster is flagged as high using z_AB with no covariates; 0 otherwise

flag_SQ_noc = 1 if a cluster is flagged as high using z_SQ with no covariates; 0 otherwise

flag_BP_noc = 1 if a cluster is flagged as high using z_BP with no covariates; 0 otherwise

flag_AB_cov = 1 if a cluster is flagged as high using z_AB with covariates; 0 otherwise

flag_SQ_cov = 1 if a cluster is flagged as high using z_SQ with covariates; 0 otherwise

flag_BP_cov = 1 if a cluster is flagged as high using z_BP with covariates; 0 otherwise

p_val = observed proportion of readmissions in the validation data

high = zip code considered high on the basis of p_val

To run the file, put the .do file and the two data files into a common directory and modify the line in the code (line 6)

local directory="C:\Users\CMcCulloch\Documents\Temp"

by replacing the location within the quotes (C:\Users\CMcCulloch\Documents\Temp) with the name of that directory. The simulation-based approach runs slowly because of the large number of replications used. If you want it to run faster, modify the line in the code (line 277)

local nreps=500000

to use a smaller number of replications, e.g., “local nreps=10000”. If you do not have Stata, the .do file is a text file that you can view with any text editor.

We have also included a file (“flagging plot big modified results check.csv”) so you can check your run of the code with ours to make sure you obtained the same results.

Finally, we also include four data sets with differences in mean square error of prediction and flagging rates for logistic and Poisson models.
