# Supplementary figures and images for: Improved prediction and flagging of extreme random effects for non-Gaussian outcomes using weighted methods
Source: Biometrics. 2025 Jul 26;81(3):ujaf094. doi: 10.1093/biomtc/ujaf094 (PMC12309285; doi:10.1093/biomtc/ujaf094)

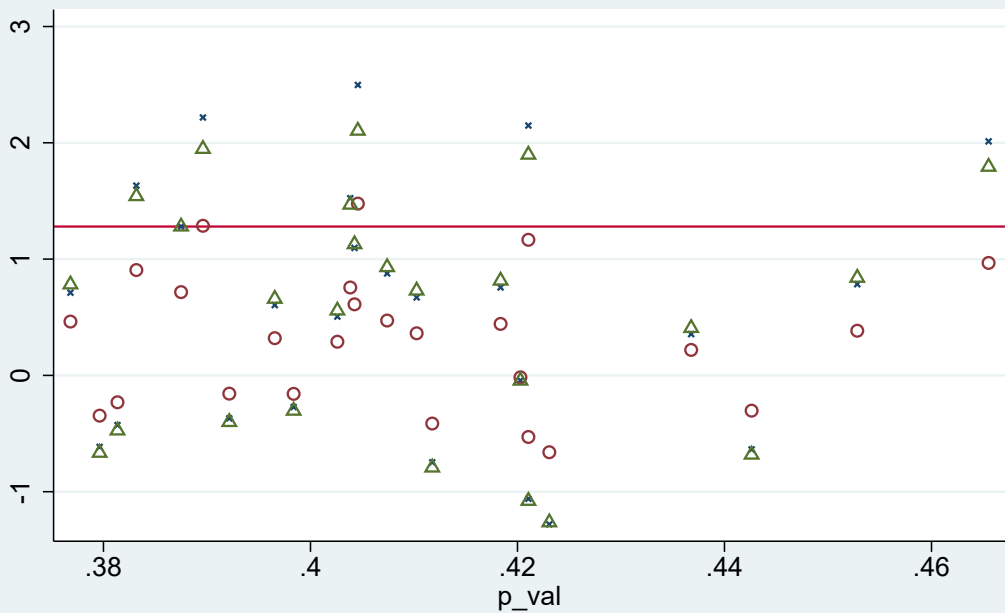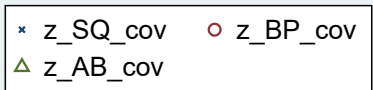

Supplement: ujaf094_Supplemental_Files — Web Appendices, Tables, and Figures referenced in Sections 2.2, 4.1, 4.2, and 5, as well as data and code to implement the algorithms in Section 3 are available with this paper at the Biometrics website on Oxford Academic. [file ujaf094_supplemental_files.zip › Supplemental_data_asthma_example/flagging plot big modified_cov.pdf]

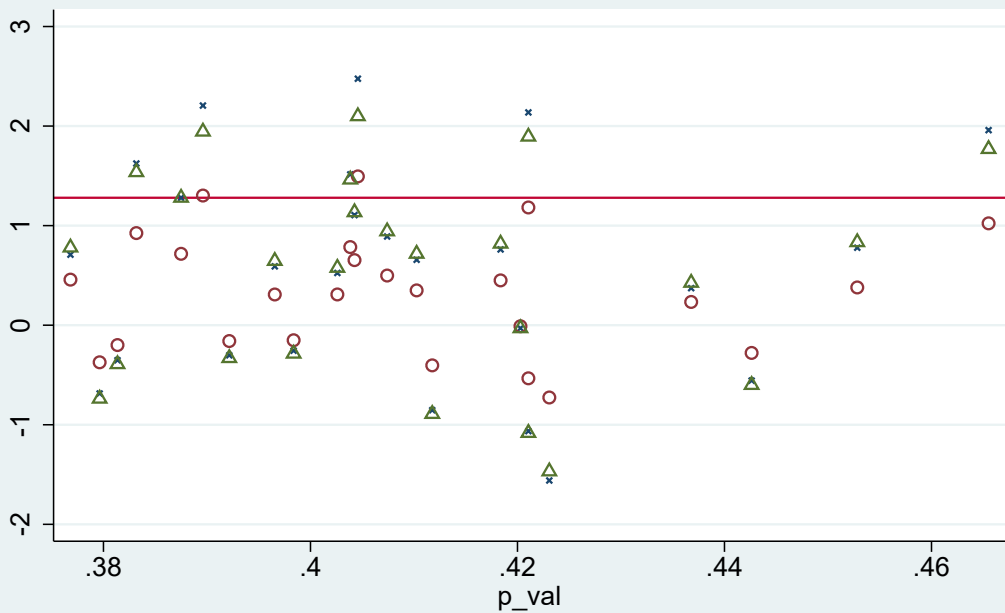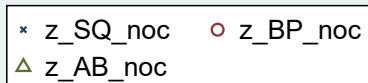

Supplement: ujaf094_Supplemental_Files — Web Appendices, Tables, and Figures referenced in Sections 2.2, 4.1, 4.2, and 5, as well as data and code to implement the algorithms in Section 3 are available with this paper at the Biometrics website on Oxford Academic. [file ujaf094_supplemental_files.zip › Supplemental_data_asthma_example/flagging plot big modified_noc.pdf]
